# Isolation and characterization of a protective monoclonal antibody targeting outer membrane protein (OmpA) against tuberculosis

**DOI:** 10.1128/spectrum.02942-24

**Published:** 2025-02-18

**Authors:** Huoming Li, Jiahong Ji, Mengjin Qu, Xiuling Ma, You Zuo, Minghui Tang, Lingyuan Zeng, Hao Li

**Affiliations:** 1 National Key Laboratory of Veterinary Public Health and Safety, College of Veterinary Medicine, China Agricultural University 34752 , Beijing, China; 2 Key Laboratory of Veterinary Public Health of Ministry of Agriculture, State Key Laboratory of Veterinary Biotechnology, Harbin Veterinary Research Institute, Chinese Academy of Agricultural Sciences 34752 , Harbin, China; University of Hawaii at Manoa, Honolulu, Hawaii, USA

**Keywords:** tuberculosis, humoral immunity, monoclonal antibody, OmpA, phagocytosis

## Abstract

Tuberculosis (TB) caused by *Mycobacterium tuberculosis* (Mtb) complex is an important zoonotic infectious disease around the world, and the One Health approach is an essential strategy for TB prevention and control. The abundant surface antigens present on the cell wall of Mtb can induce protective antibodies; however, its full characterization remains incomplete. Previous research has shown that antibodies targeting surface proteins can enhance host defense mechanisms. Therefore, identifying antigens that stimulate the production of protective antibodies is essential. In this study, we focused on a select antigen, outer membrane protein (OmpA), located on the outer membrane of Mtb. By screening for antibodies with protective effects in phagocytosis and intracellular killing *in vitro*, we identified a protective antibody targeting OmpA. We prepared an antibody, designated as 1E1, belonging to the IgG2b isotype, which exhibited high titers of 1:2,048,000. Further research demonstrated that antibody-induced protection was achieved by promoting opsonophagocytosis in a dose-dependent manner, enhancing phagosome-lysosome fusion, and inhibiting mycobacterial intracellular growth. These findings were corroborated *in vivo*, with a reduction in bacterial loads of approximately 0.7 log observed in the preventive group and almost 1.0 log in the therapeutic group of mice treated with the antibodies, compared to the negative control group. Cytotoxicity assays, animal toxicity analyses, and pharmacokinetic evaluations confirmed the safety and sustained effectiveness of the antibody *in vivo*. These findings indicate that OmpA can elicit protective antibodies and may serve as a treatment strategy for drug-resistant TB and a promising antigen for TB vaccine development.

**IMPORTANCE:**

In this study, we identified a protective antibody targeting the outer membrane protein (OmpA) of *Mycobacterium tuberculosis*. This monoclonal antibody (MAb) belongs to the IgG2b isotype and exhibits high titers of 1:2,048,000 to the antigen. The cell infection assays demonstrated that antibody protection was achieved by promoting opsonophagocytosis in a dose-dependent manner, enhancing phagosome-lysosome fusion, and inhibiting mycobacterial intracellular growth *in vitro* and *ex vivo*. Cytotoxicity assays, animal toxicity analyses, and pharmacokinetic evaluations confirmed the safety and sustained effectiveness of the antibody *in vivo*. Furthermore, the mAb 1E1 can reduce the organs' bacterial burdens and pathological damages in the prevention mouse model as well as the treatment models. Above all, in this study, we found a novel mAb named 1E1 with IgG2b isotype targeting OmpA can have protection against tuberculosis (TB) in mice, which may serve as a treatment strategy for drug-resistant TB and a promising antigen for TB vaccine development.

## INTRODUCTION

Tuberculosis (TB) is currently one of the most lethal infectious diseases around the world (1). TB as a zoonotic disease causes millions of deaths annually and poses a serious economic burden, particularly in low-income countries (2). *Mycobacterium bovis* (*M. bovis*), part of the *Mycobacterium tuberculosis* (Mtb) complex, is associated with bovine TB in humans and animals. Traditionally, antibiotics were considered an effective and cost-efficient means to control TB. The extensive use of antibiotics has advanced the therapeutic management of TB. However, the emergence of multidrug-resistant tuberculosis (MDR-TB) and extensively drug-resistant TB (XDR-TB) has posed significant challenges in TB diagnosis and treatment (3). In light of the current limitations in drug resistance, there is growing interest in exploring the potential antibodies with strong prophylactic and therapeutic effects as reasonable alternatives.

The lack of an effective anti-TB vaccine remains a major barrier to the treatment and elimination of TB. *Bacillus Calmette-Guerin* (BCG) is currently the only vaccine approved for universal vaccination and was developed a century ago (4). However, its efficacy in preventing TB in adults is not optimal. It is becoming increasingly clear that a more effective TB vaccine is needed. Bovine TB symptoms are clinically like those of human TB, and the *M. bovis g*enome is 99.9% or more homologous to the Mtb genome (5, 6). Moreover, *M. bovis* has been used widely for establishing animal infection models, and evaluating protective immune responses and TB vaccine efficacy (7–9). Several studies have verified that antibodies play a protective role in preventing and treating TB infections (10–13). These studies support the antibodies’ protection roles against TB and antibodies as an alternative strategy to treat TB.

The surface antigens of Mtb play versatile roles during infection, including adhesion, phagocytosis, intracellular survival of macrophages, and antigen presentation by dendritic cells (14). Antibodies generated against surface antigens were involved in many bacterial infection processes and were regarded as excellent therapeutic targets (7, 12). Among surface antigens, OmpA belongs to a genetically related group of surface-exposed porin proteins with high copy numbers in the outer membrane protein of bacteria (15). Human antibodies targeting the porin domain of *Escherichia coli* OmpA induced bacterial aggregation but did not alter the overall bacterial burden in mice (16). An anti-Omp34 antibody with a high titer was able to eradicate *Acinetobacter baumannii* from the livers and spleens of mice, enhancing their survival rate (17). Additionally, a monoclonal antibody (MAb) targeting *A. baumannii* OmpA inhibited biofilm formation, adhesion, and proliferation in A549 epithelial cells (18). The protein encoded by Rv0899 of Mtb, named OmpA, shares 65% identity with *E. coli* OmpA (19, 20). Mtb OmpA not only stimulated the release of IFN-γ but also inhibited the growth of bacteria in macrophages and mice (21). The role of antibodies induced by OmpA in anti-TB remains to be elucidated.

In this study, the OmpA protein was expressed, purified, and used for mouse immunization for antibody production. MAbs targeting OmpA were isolated using hybridoma technology and purified. One MAb, named 1E1, was isolated and shown to inhibit bacterial infections in cell and mouse models. This study provides opportunities for TB prevention and treatment and paves the way for novel vaccine development.

## MATERIALS AND METHODS

### Bacterial and cell lines

All the strains used in this study were stored in our laboratory. *Mycobacterium bovis* (*M. bovis*) C68004 and *M. bovis* BCG Pasteur 1173P2 strain were cultured in 7H9 medium with OADC (BD) at 37°C. All cells used in this study, including SP2/0 myeloma tumor cells, J774a.1, and A549 cells, were cultured in DMEM supplemented with 10% fetal bovine serum at 37°C in a humidified atmosphere containing 5% CO_2_.

### Animal studies and ethics statements

All animals and cell experiments involving virulent strains were conducted at the Biosafety Level-3 (BSL-3) Laboratory at China Agricultural University. Animal experiments were approved by the Experimental Animal Welfare Ethical Review Consent of China Agricultural University (license no. AW10704202-2-1). Female C57BL/6J mice (6–8 weeks old) were purchased from Si Bei Fu (SPF) Bio-Technology Company (Beijing, China). The mice’s blood was collected from the venous sinus or via cardiac puncture, and the cervical dislocation method was used to euthanize them.

### OmpA protein preparation

Polymerase chain reaction (PCR) was done with forward primer (OmpA-F: 5′-CCG*GAATTC*GAGCGGCCCCAGTCCGTTAC-3′, EcoR I) that bind to the 150 bp site of OmpA nucleotide sequence and reverse primer (OmpA-R: 5′-CCC*AAGCTT*GTTGACCACGATCTCGACGCGAC-3′, Hind III), from BCG chromosomal DNA. In this study, the pET28a (+) vector with *E. coli* BL21 was used to express OmpA. Briefly, the product of OmpA was cloned into the pET28a (+) vector with restriction sites EcoR I and Hind III, generating recombinant protein with N- and C-terminal His_6_-tag. The plasmid was then transformed into BL21 (DE3) cells for affinity purification. Overnight cultures of recombinant *E. coli* strains were inoculated (1:100) into fresh Luria-Bertani (LB) broth medium and grown at 37°C for 3 h to an OD_600_ of 0.5, at which point the culture was induced with 1 mM isopropyl β-d-1-thiogalatopyra-noside (IPTG) for 3 h. Bacterial cells were collected by centrifuging at 5,000 rpm for 10 min, followed by washing three times with phosphate-buffered saline (PBS). The pellets were suspended in lysis (LE) buffer (50 mM Na_2_HPO_4_, 0.3 M NaCl, pH 8.0) and disrupted with 15 min of sonication. The membrane and insoluble components were cleared from the lysates using 12,000 rpm centrifugation for 20 min. Proteins in the cleared lysate were loaded onto a nickel-nitrilotriacetic acid (Ni-NTA) affinity chromatography column (Genscript, Nanjing), followed by elution with successively higher concentrations of imidazole (100–500 mM). Protein concentration was measured using the bicinchoninic acid (BCA) assay (Solarbio).

### Production and characterization of OmpA antibodies

The MAbs were generated using hybridoma technology. Briefly, three female BALB/c (6–8 weeks) were subcutaneously immunized three times with purified OmpA (1 mg/mL) on the back of the neck (100 µg purified protein with an equal volume of aluminum adjuvant (Thermo Fisher, China) at 2-week intervals. Serum titers were determined by indirect enzyme-linked immunosorbent assay (ELISA), using purified OmpA as an antigen. After the anticipated serum titers (>1 × 10^5^) were reached, the splenocytes were fused with SP2/0 myeloma cells. The fused cells were cultured on HAT medium, and the positive monoclonal hybridoma lines were screened for reactivity with the OmpA protein. Among the three isolated MAbs, one Mab named 1E1, which showed strong reactivity, was selected for further studies. To acquire MAbs, ascites were produced by injecting hybridoma cells into mice via the intraperitoneal route, and antibodies were purified using a protein-G column (Genscript, China). The purity and specificity of the MAbs were assessed by SDS-PAGE and Western blot.

### ELISA

The 96-wells plates (Costar, USA) were coated with purified OmpA protein (0.1 µg/well) and incubated overnight at 4°C. Subsequently, individual hybridoma culture supernatants or purified MAbs (1:5,000) were added to each well (100 µL/well), followed by incubation with horseradish peroxidase (HRP)-conjugated goat anti-mouse IgG (1:10,000) (CWBIO, CW0102S). The negative control consisted of the serum from mice injected with PBS. The optical density of (substrate) was measured at 450 nm using a multifunctional microplate reader (Spark). A positive well was deemed if the OD_450 nm_ value of the lowest dilution well was 2.1 times higher than that of the negative control well (22).

For whole-bacterial ELISA, BCG and *M. bovis* cultured in 7H9 medium with or without Tween 80 for 2 weeks, were collected by centrifugation at 5,000 rpm for 10 min. After being washed three times with PBS, the bacterial pellets were inactivated by heating at 80°C for 2 h. Bacterial cells were suspended in distilled water and dispersed using a 25-gauge needle syringe through passage 15 times to obtain a single bacterial suspension. The concentration of the bacterial suspension was adjusted to 1 × 10^8^ CFU/mL and coated with 100 µL/well in 96-well ELISA plates, followed by drying at 56°C. The −20°C pre-cooled methanol was added to each well and incubated for 2 h at 4°C. After an additional washing step, 5% skimmed milk was used to block for 2 h, and then the process was performed as indicated above.

To determine the antibody isotype, a MAb isotype kit (Frdbio, China) was used, by the manufacturer’s instructions. Briefly, the purified OmpA protein was coated in 96-well plates with 0.1 µg/well and incubated overnight at 4°C. The culture supernatant from the hybridoma-secreted antibody was added to each well (100 µL/well) and incubated for 1 h at 37°C. HRP-conjugated isotype secondary antibody was diluted (1:500) with a phosphate buffer solution containing 0.5% Tween-20 (PBST) and incubated for 1 h at 37°C. The antibody isotypes were determined based on the corresponding positive wells.

### Phagocytosis assay

The effects of MAbs on phagocytosis and bacterial invasion were assessed using flow cytometry, as previously described with some modifications (23–25). Briefly, J774a.1 cells (1 × 10^5^ cells/well) were cultured overnight in 24-well plates. The bacteria were stained with 1 mg/mL FITC dissolved in 0.1M carbonate/bicarbonate buffer (PH 9.5) for 2 h on a shaker at 37°C and 90 rpm. After staining, the bacteria were washed three times with PBS and disrupted 15 times using a 25-gauge needle to obtain a single-cell bacterial suspension. Mab and 1E1 (2, 10, and 100 µg/mL) were separately mixed with BCG-FITC (2 × 10^7^ CFU) and incubated for 1 h at 37°C. The bacteria were then washed three times with PBS and resuspension with DMEM.

After the cells were washed, they were infected with BCG-FITC (MOI = 10:1 or 1:1) for 3 h. Extracellular bacteria were removed by washing three times with PBS. Macrophages were fixed with 4% paraformaldehyde (PFA) for 30 min at room temperature and resuspended in 500 µL 5% FCS/PBS. Finally, cell samples were analyzed using a BD Fortessa cytometer. The data were analyzed using the FlowJo software.

### Intracellular bacterial killing assay

The J774a.1 cells (1 × 10^5^ cells/well) were cultured overnight in a 24-well plate. PBS-washed BCG (1 × 10^7^ CFU) was incubated with 1E1 or mGO53 (negative control mAb) at a final concentration of 50 µg/mL for 1 h at 37°C. The coated cells were infected with bacteria (multiplicity of infection [MOI] = 10:1) for 3 h, and extracellular bacteria were removed by washing three times with PBS. All cells were then incubated with 0.1% Triton X-100 at 37°C for 10 min. The bacterial solution was serially diluted, plated on the 7H10 complement with 10% OADC, and incubated for 3 weeks at 37°C.

### *Ex vivo* mycobacteria growth inhibition assay

Blood samples collected from anesthetized C57BL/6J (6–8 weeks) via cardiac puncture were drawn into CPT vacutainer tubes (BD, 362761), and peripheral blood mononuclear cells (PBMCs) were isolated. Briefly, the blood samples were diluted 1:1 with RPMI 1640 (Gibco) and carefully layered over the surface of Mouse Lymphocyte Isolation Solution (Beyotime, C0029S), followed by centrifugation without the brake applied at 500 × *g* for 30 min at 20°C. PBMCs were transferred to a new tube after carefully removing the upper plasma layer and washed three times using PBS with 250 g for 10 min.

PBMCs (3 × 10^5^ cells/well) were suspended in PBS and distributed into 24-well plates, and subsequently exposed to PBS-washed BCG (3 × 10^6^ CFU) or *M. bovis* (3 × 10^6^ CFU) incubated with 1E1 at concentrations of 5, 50l, and 250 µg/mL. The mAb mGO53 was used as the negative control (26–28). All wells were cultured at 37°C for 4 days and subsequently treated with 0.1% Triton X-100 at 37°C for 10 min. Dilutions of BCG or *M. bovis* were plated onto 7H10 agar supplement with 10% OADC and cultured at 37°C for 3 weeks until the individual colonies were observed.

### Confocal microscopy

The J774A.1 (1 × 10^5^) cells were cultured overnight on coverslips within 24-well plates. Bacteria stained with FITC at a concentration of 1 mg/mL were washed three times with PBS, and then these bacteria were incubated with mGO53 or 1E1 at the final concentration of 50 µg/mL. The bacteria were used to infect the cells for 3 h at an MOI of 10:1. Following three washes with pre-warmed PBS, the lysosome marker, Lyso-Tracker Red (Beyotime Biotechnology, C1046), was added to the cells, which were then incubated at 37°C for 1 h. The cells were fixed with 4% PFA for 30 min at room temperature. Confocal microscopy (Nikon A1HD25) was performed following blocking with an anti-fluorescence quenching reagent (Beyotime Biotechnology, P0133) to assess the extent of phagosome-lysosome fusion. To evaluate the number of phagosome-lysosome fusions, confocal microscopy was utilized to randomly capture 10 distinct images at a magnification of ×100 (oil immersion lens). The total number of cells and BCG phagosomes (defined as BCG colocalized with lysosomes) within the field of view were quantified. Subsequently, the number of BCG phagosomes was determined to quantify the percentage of phagosomes that colocalized with lysosomes, as indicated by LysoTracker staining.

Colocalization of lysosome with BCG (%) $=\left[\sum_{i-1}^{n}PS÷\sum_{i-1}^{n}TC\right]\times 100\%$ (*n* = 10)

where PS refers to the total number of BCG phagosomes, and TC refers to the total number of cells in 10 distinct images.

### Cytotoxicity assay, pathological, and pharmacokinetic evaluation

The cytotoxicity of MAbs was assessed *in vitro* using the CCK-8 assay (Solarbio, CA1210). Briefly, J774A.1 cells (1 × 10^4^) were cultured in 96-well plates for 24 h. PBS and varying concentrations of MAbs (1, 5, 50, 100, 250, and 500 µg/ml) were added to the wells, with each concentration tested in quintuplicates, and the cells were incubated for an additional 24 h. Subsequently, 10 µL CCK-8 reagent was added to each well and the plates were incubated for 2 h before measuring the optical density at 450 nm (OD_450_).

To evaluate the persistence of MAbs in circulation, 500 µg of MAbs was administered intraperitoneally (i.p) to mice. Blood samples were collected from three mice at the retroorbital sinus on days 0, 3, 6, 9, 12, 15, 18, 21, 24, 27, and 30. The levels of residual MAbs were quantified using ELISA techniques on serum prepared from the collected blood samples. Additionally, to evaluate whether antibody-mediated immunity induced any adverse effects on the organs, the lungs, and spleens from the same cohort of mice were subjected to pathological analysis 7 days post-MAb injection.

### Passive immune protection

To evaluate the protective efficacy of MAbs *in vivo*, all C57BL/6J female mice in this experiment were maintained under BSL-3 conditions for about a week for adaptive feeding. Then, these mice were divided into a prevention group and a treatment group. In 24 h after infection, three of the PBS control mice were sacrificed for evaluation of the initial infection dose, which was verified as between 100 and 200 CFUs per mouse for all batch experiments.

The prevention group experiments were performed as previously described. Briefly, each mouse received 500 µg of MAbs or equal volume PBS via intraperitoneal injection 5 h before *M. bovis* infection. Each mouse was inoculated with 200 CFU of *M. bovis* via the intranasal (i.n.) route. After 14 days, the mice were euthanized, and lung homogenates were plated on 7H10 agar supplemented with 10% OADC (BD) and incubated at 37°C for 3–4 weeks until bacterial colonies developed. Bacterial counts were performed to assess the organs’ bacterial burden.

Similarly, each mouce in the therapeutic group was first infected with 200 CFU of *M. bovis*. After 24 h, the mice were administrated either 500 µg/mouse of MAbs via intraperitoneal injection once a week for four times, while the control group received only 500 µL of PBS. The mice were euthanized and necropsied to evaluate the therapeutic effect of the antibody after last administration. The right lungs from different mice were homogenized and coated in 7H9 plates for 3 weeks to count the lung burdens.

### Histopathological analysis

The tissue from different mice was PFA-fixed for 1 week to analyze pathological lesions. Briefly, lungs and spleens from both the MAb-immunized and PBS-immunized groups were fixed in 10% formalin solution for 7 days. All tissues were embedded in paraffin and sectioned into 3-µm-thick slices. The organs of five mice in each group were sent for section preparation. All sections were stained with hematoxylin and eosin (H&E) (Solarbio, G1120) after mounting on the glass slides and de-paraffinization. The Hamamatsu NanoZoomer-S60 digital slide scanner was employed to scan all tissue slides. Five microscopic fields from *n* = 5 mice per group for each organ section were selected for histopathological analysis. The resulting images were analyzed for various pathological features, including inflammation, alveolar septa deformation, and bronchial mucus hypersecretion, and an expert in pathology who was blinded to the experiment gave a pathological score for each section (22, 29).

### Antibody homology modeling and antibody-antigen docking

The 1E1 Fv model was constructed using Discovery Studio (DS) through a three-step process, involving the Identification of the Framework Template, Modeling of Antibody Framework, and Antibody Loop Modeling. Specifically, the Identification of Framework Template was utilized to search for a template structure suitable for both VH and VL domains of the 1E1 antibody. Complementarity-determining region (CDR) loops were modeled using the Model Antibody Loops module following the initial 1E1 antibody model. The structural model of OmpA was predicted using the Alphafold 3 online server (https://golgi.sandbox.google.com/).

Antibody-antigen docking involving both the 1E1 antibody model and the OmpA structure was performed using the DS software 2019 package. Rigid-body docking was conducted using ZDOCK with an angular step size of 15° and a distance cutoff of 10 Å. Process poses were generated utilizing the CDR3 of VH and VL domains, while non-CDR3 residues of the antibody were masked. Subsequently, RDOCK was employed to refine the docked poses.

### Statistical analysis

Statistical analyses were performed using GraphPad Prism 9.5 software. ELISAs were conducted in triplicate, and the statistical data were presented as means. Significant differences between the MAb treatment group and the control group were assessed using either unpaired one-way analysis of variance (ANOVA) or Student’s *t* test. Results were interpreted as follows: ns: not significant; *P < 0.05; **P < 0.01; ***P < 0.001; and ****P < 0.0001.

## RESULTS

### Preparation, purification, and characterization of MAbs

**Fig 1:**
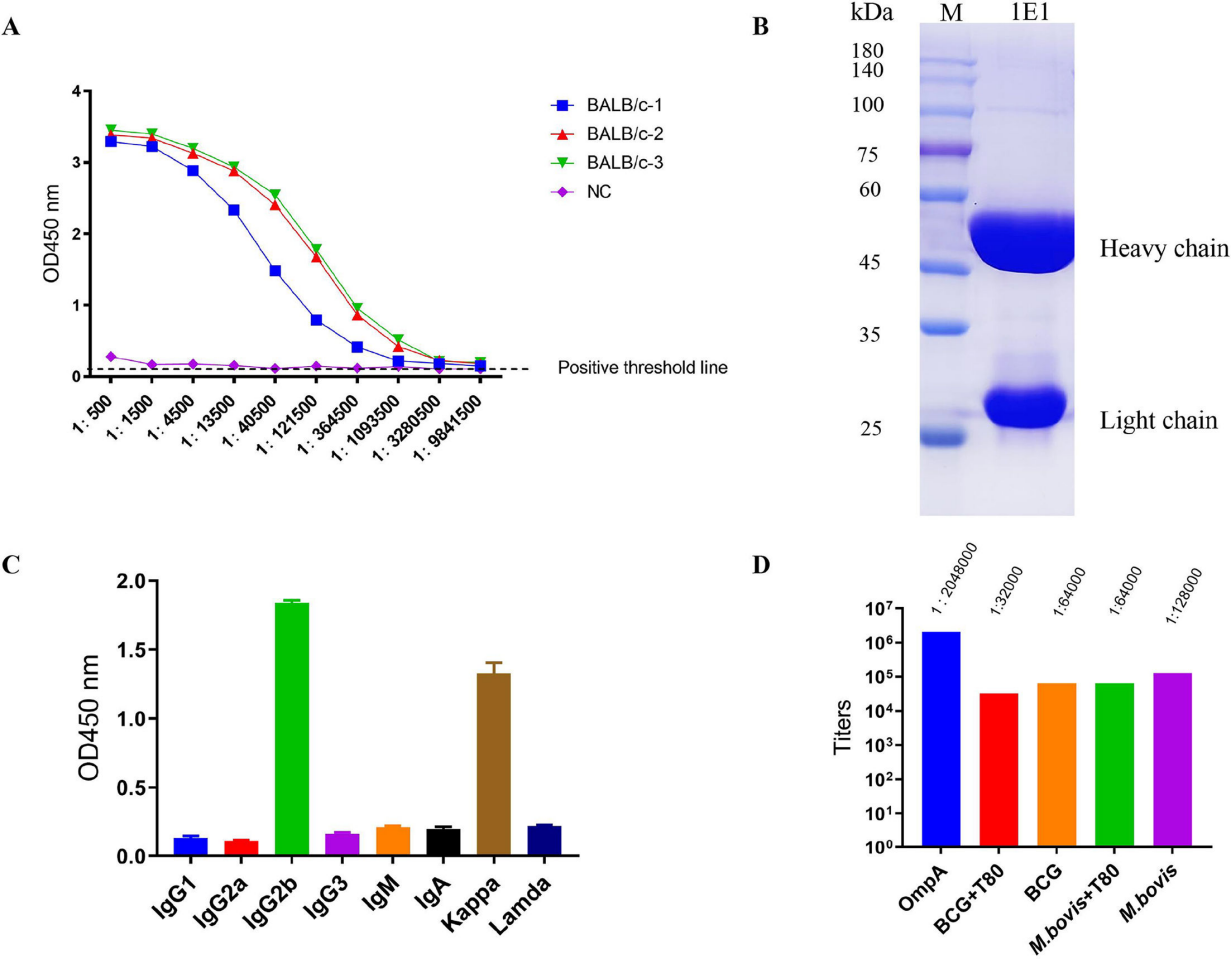
Purification and identification of anti-OmpA monoclonal antibody 1E1. (**A**) The serum titers of immunized- mice after three immunizations with 100 µg purified OmpA mixed with an equal volume of aluminum adjuvant per mouse for 2-week intervals. After 6 weeks, the serum samples were collected by orbital blood sampling, and the serum efficacy was determined, with 1:500 dilution followed by threefold dilution. The positive well was defined as the target well value that was higher than twofold PBS-immunized mice serum. (**B**) The purified Mab 1E1 was displayed on the SDS-PAGE with a Prestained 180 kDa Protein Ladder. (**C**) The Mab’s isotype was analyzed by ELISA. Shown is one representative of three independent biological replicates. (**D**) The MAbs titers targeting the antigen or mycobacterial strains were determined. The ELISA plate coated with heated-inactivated BCG (1 × 10^8^/well) or *M. bovis* (1 × 10^8^/well) was incubated with serial dilution antibody for 1 h at 37°C, followed by incubation with goat anti-mouse secondary antibody labeled with HRP. The OD_450 nm_ was measured and antibody titers were determined. T80: Tween-80 reagent. The experiment was repeated three times, and the representative data from one replicate are shown.

OmpA protein was expressed and purified by the *E. coli* expression system. The purity and specificity of the protein were confirmed through SDS-PAGE Coomassie Brilliant Blue staining and Western blot (Fig. S1). Following immunization of three BALB/c mice with 100 µg/mL purified OmpA protein, serum titers reached 1.0935 × 10^6^ (Fig. 1A). Hybridoma cell lines producing stable antibodies were successfully generated using hybridoma technology in this study. The light and heavy chains of purified MAb 1E1 were analyzed by SDS-PAGE (Fig. 1B). Additionally, the antibody isotype was identified as IgG2b (Fig. 1C).

Specific antigen recognition was essential for antibody function. Titers of IE1 against purified OmpA, BCG, or *M. bovis* were determined using whole bacterial ELISA (Fig. 1D). Compared to mycobacterial strains, these titers were significantly higher at 1:2,048,000 for purified OmpA protein. Furthermore, the 1E1 antibody showed higher affinity toward both purified OmpA protein and all mycobacterial strains, including BCG (1:64,000) and *M. bovis* (1:128,000), except OmpA-negative *M. smegmatis*. The addition of Tween-80 reduced the binding ability of antibodies toward bacteria. These findings collectively indicate that the antibody specifically binds to Mycobacterial species expressing OmpA.

### MAbs exhibit promoted effects on the phagocytosis and intracellular killing of BCG *in vitro*

**Fig 2:**
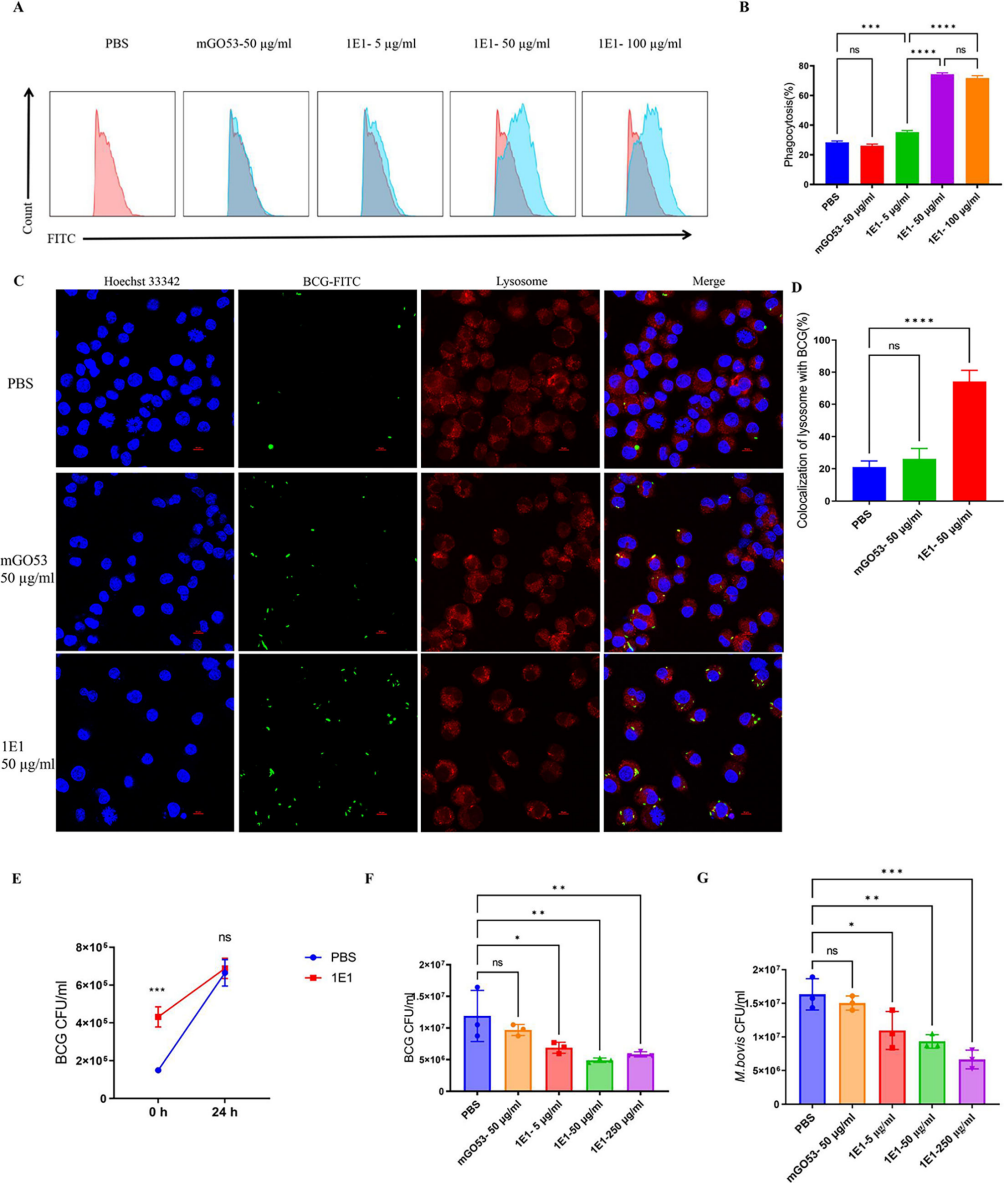
MAb 1E1 can increase macrophage phagocytosis and have inhibitory effects against mycobacterial infections. (**A**) Flow cytometry was used to determine the phagocytosis of BCG-FITC in J774a.1 cells. (**B**) Statistical results of phagocytosis experiments by flow cytometry were shown. (**C**) The colocalization of BCG with phagolysosomes was analyzed by confocal microscopy. J774a.1 cells were infected by BCG-FITC (MOI = 10:1) after BCG was incubated with PBS or 1E1 for 3 h. Scale bar, 20 µm. (**D**) The percentages statistics of colocalization of lysosome with BCG in the experiment were presented. At least 300 cells in 10 fields were counted in each group. (**E**) The growth inhibition was shown. The J774a.1 were infected with BCG (MOI = 10 : 1) for 3 h. Then, the cells were washed with pre-warmed medium to remove extracellular bacteria, and the point was counted as 0 h. The cells were lysed and harvested for CFU counting, and the intracellular bacteria were recovered at 0 and 24 h. (**F**) and (**G**) The PBMCs (3 × 10^5^ cells/well) from C57BL/c were infected with BCG (MOI = 10:1) or *M. bovis* (MOI = 10:1) incubated with PBS, 5, 50, or 250 µg/mL for 4 days. The bacterial survival was counted and the results (*n* = 3) were shown The bacterial survival was determined in triplicates. The results were shown as mean values ± SD and were analyzed by one-way ANOVA analysis. *P < 0.05; **P < 0.01; ***P < 0.001; and ****P < 0.0001; ns, no significant. All experiments were repeated three times (biological replicates), and the representative data from one replicate are shown.

The opsonic functions of antibodies elicited by OmpA were further investigated to determine their role in the humoral immune response against TB infection *in vitro*. BCG-conjugated FITC was incubated with various concentrations of MAbs before infecting macrophage cells. The 1E1 antibody was found to enhance phagocytosis of BCG-conjugated FITC in a dose-dependent manner compared to PBS, as shown in Fig. 2A and B. And the inhibitory effects of macrophage phagocytosis were further proved by the plate assay (Fig. 2E). Furthermore, the frequency of lysosome colocalization with BCG was significantly higher following incubation with 1E1 antibody compared to the PBS and mGO53-treated groups (Fig. 2C and D). These results indicated that the 1E1 antibody has the potential to enhance phagocytosis and phagosome-lysosome fusion.

To assess the intracellular inhibitory effects on mycobacteria, PBMCs from C57BL/6J were also infected with BCG or *M. bovis*, co-incubated to specified times, and then harvested for bacterial enumeration. The results indicate that significant mycobacterial growth was inhibited in PBMC cells (Fig. 2F and G), which further supports that the 1E1 antibody has protection effects against mycobacterial infections.

### Cytotoxicity assay, pathological analysis, and pharmacokinetic evaluation of the MAbs

**Fig 3:**
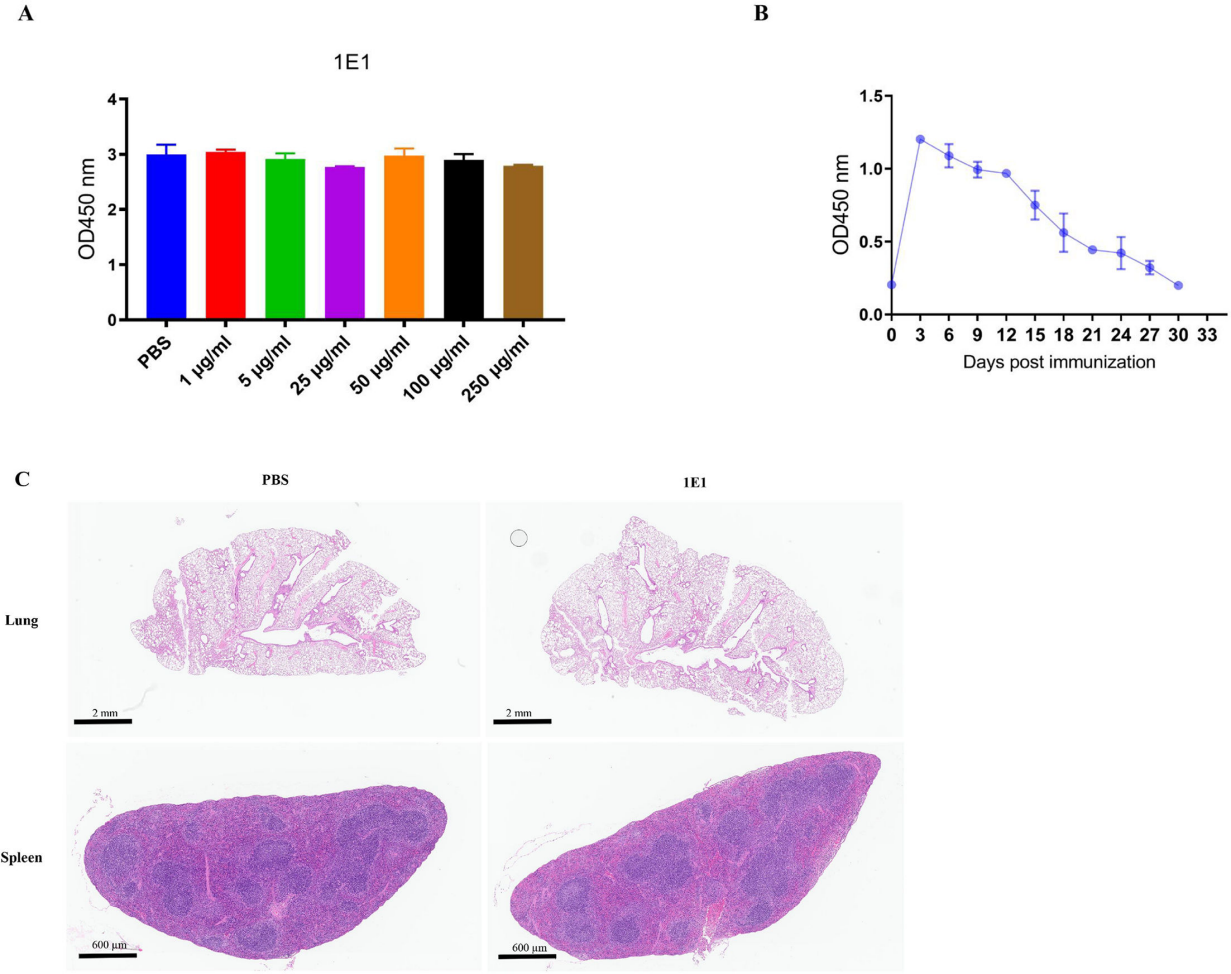
Safety and pharmacokinetic analysis of 1E1. (**A**) The cytotoxicity was measured after J774a.1 cells were co-cultured with a series of different concentrations of antibodies. (**B**) The antibody persistence in mice. The blood samples were taken from the retroorbital sinus at 3-day intervals after 500 µg MAbs administration. The titers of MAbs were measured by ELISA. (**C**) Pathological analysis was performed by H&E staining. Representative pathological images of lung and spleen from mice injected with 1E1. The scale bar is set at 600 µm in the spleen and 2 mm in the lung. The images were annotated using the Aperio ImageScope (Leica Biosystems).

To assess the cytotoxicity of 1E1 on the mammalian cell, the survival rate of J774A.1 at series MAbs concentration was measured by OD_450 nm_. The 1E1 did not exhibit significant cytotoxicity even at 250 µg/mL concentration as shown in Fig. 3A.

The protective function of antibodies depends on their persistence *in vivo*. The antibody half-life in the bloodstream was evaluated by administering 500 µg of MAbs intraperitoneally, and antibody titers were monitored on days 0, 3, 6, 9, 12, 15, 18, 21, 24, 27, and 30 post-immunizations. Figure 3B depicts the antibody titers over time in the bloodstream. The results showed that the serum antibody peaked at day 3 and followed a gradual decline over the following days. Additionally, a comparative analysis of spleen and lung pathology images from PBS-immunized and 1E1-immunized mice showed no discernible tissue damage due to antibody immunization (Fig. 3C). Furthermore, behavioral changes in immunized mice, such as appetite, hair, and diarrhea, did not exhibit any abnormal condition during the 30-day observation period.

### Protective effects of the MAbs *in vivo*

**Fig 4:**
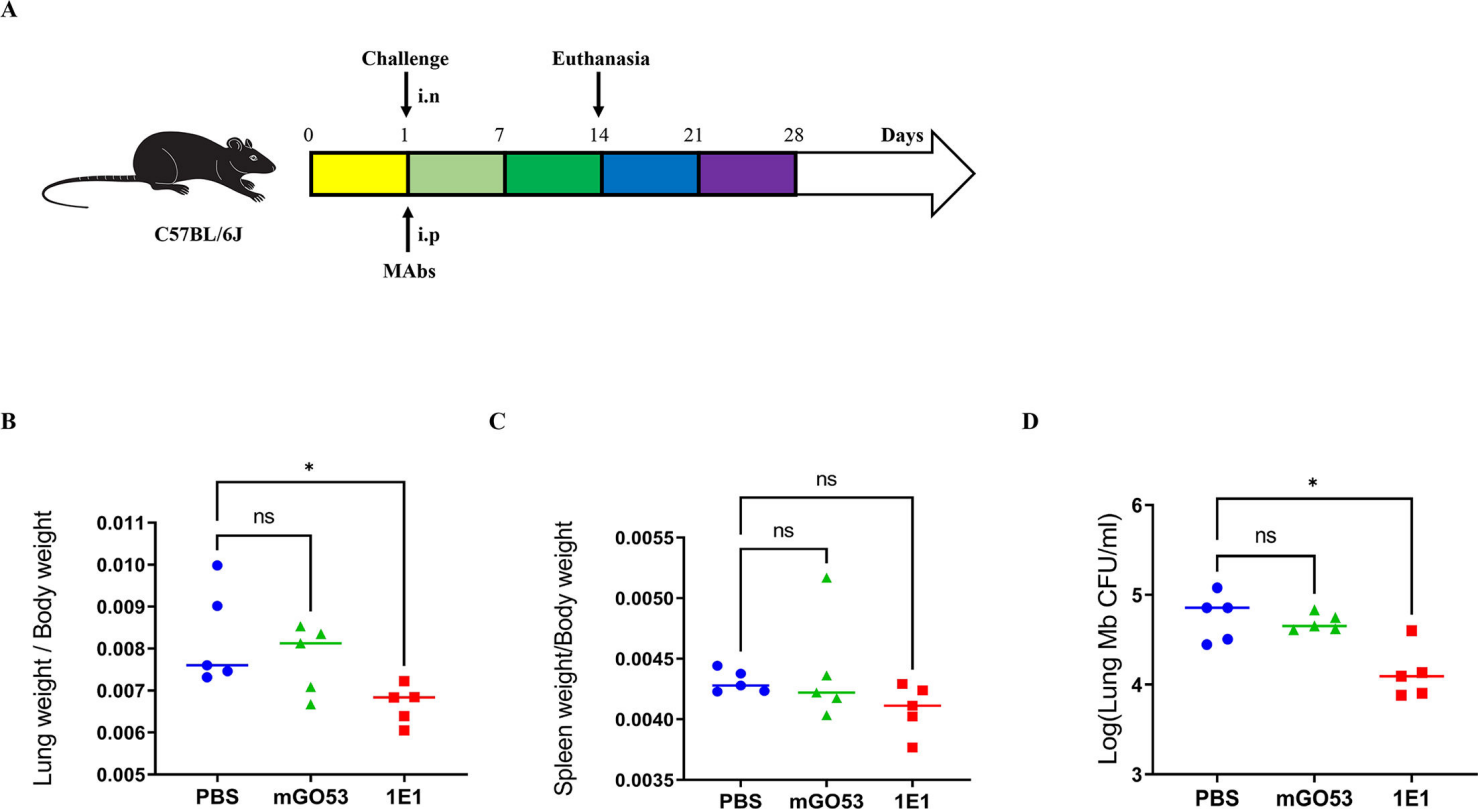
Anti-OmpA Mab 1E1 inhibits *M. bovis* in C57BL/c. (**A**) Experimental process for evaluating the preventing effect of MAbs in mice. (B–D) The organ coefficients of the lung (**B**) and spleen (**C**) and the number of viable bacteria in the lungs (**D**) were determined. Activity of anti-OmpA MAbs: A1E1 (red dot), PBS (blue dot), and control group (green dot). Mice were injected intraperitoneally with 500 µg of mGO53 or 1E1 respectively, or 500 µL PBS, and infected with *M. bovis* via intranasal (i.n.) infection after 5 h. After 2 weeks, the mice were euthanized and lung homogenates were plated on 7H10 plates to count the bacterial load. Error bars are represented as mean ± SD. In each treatment, *n* = 5 mice. Significance was determined using a one-way ANOVA. *P < 0.05. The results are representative of three independent experiments.

To evaluate the protective effects of MAbs 1E1 *in vivo,* C57BL/6J mice were administered 500 µg of 1E1 MAbs intraperitoneally 5 h before being challenged with *M. bovis*. Behavioral changes and weights of all mice were continuously monitored for 2 weeks after challenge and treatment. A comparison of organ coefficients of lungs and spleens from mice immunized with 1E1 antibody, PBS, or control group showed that the 1E1 antibody decreased the lung index but not the spleen index (Fig. 4B and C). Lungs from all groups were used for pathological damage analysis and scored, according to Table S1, in which no significant differences were observed (Fig. S2; Table S2). Treating mice with 500 µg MAbs 1E1 resulted in an approximately 0.7 log CFU reduction in lung bacterial load compared to the control group mice mock-treat with PBS or control group (mGO53), as shown in Fig. 4D. These results are consistent with the cell assay results described above *in vitro*, further verifying that the 1E1 MAbs have protective activity against *M. bovis*.

**Fig 5:**
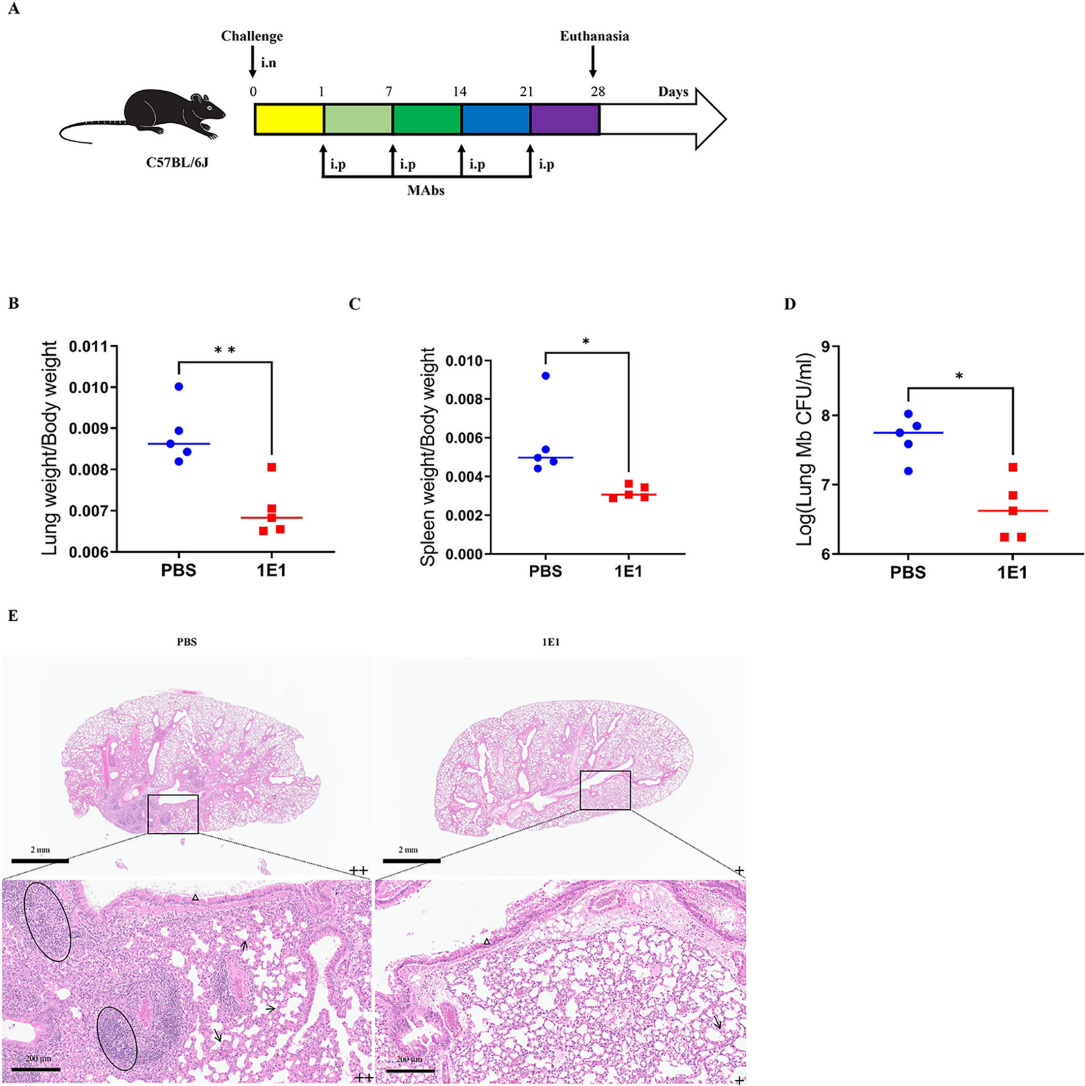
The therapeutic effect of anti-OmpA MAbs. (**A**) Experimental process for evaluating the therapeutic effect of MAbs. (B and C). The organ coefficient of the lung (**B**) and spleen (**C**) and bacterial burden of the lung (**D**). (**E**) Histopathological change in *M. bovi*s-infected mice. Each group (*n* = 5 per group) of C57BL/6 mice was infected with 200 CFU of *M. bovis* via the intranasal (i.n.) route. Following a 24-h feeding period, 500 µg of antibody or 500 µL of PBS was administrated intraperitoneally, followed by four additional injections of 500 µg of antibody or 500 µL of PBS at 1-week intervals. A necropsy was performed on the mice to evaluate the therapeutic effect of the antibody following the final administration. The lung sections exhibit perivascular and peribronchial accompanied by inflammatory cell infiltration (elliptical), alveolar septa deformation (arrows), and bronchial mucus hypersecretion (white triangle). Five fields from *n* = 5 mice per group for each organ section were analyzed. Significance was determined using a Student’s *t* test. *P < 0.05; **P < 0.01. The results are representative of three independent experiments. The extent of tissue damage was scored in the bottom right corner of each figure, with the symbols “+” and “++” indicating a progressive increase in severity.

Subsequently, the therapeutic effect of MAbs was examined on *M. bovis-*infected mice. The infected *M. bovis* mice received MAbs or PBS for four consecutive treatments via the intraperitoneal route. The organ coefficients of spleens and lungs indicated that MAb therapy attenuated the pathogenesis of *M. bovis* in mice compared to the PBS group (Fig. 5B and C). Furthermore, the bacterial loads in the MAb-treated group were also reduced by almost 1.0 log CFU compared to the PBS group (Fig. 5D). We also evaluated organ injury and inflammatory cell infiltration using H&E staining. Histopathological analysis was conducted to assess the degree of lymphocyte infiltration and other pathological lesions in lung structures based on a scoring matrix in Table S1. All the corresponding scoring values are registered in Table S3. Mice treated with 1E1 exhibited milder pathological lesions, characterized by sparse inflammatory infiltrate, slight mucus hypersecretion, and more intact lung morphology, such as alveolar septa (+). In contrast, PBS-treat mice exhibited severe lung damage, evidenced by obvious inflammatory infiltration, slight mucus hypersecretion, and alveolar septa thickening (++). Pathological observations indicated that 1E1 MAbs was protective *in vivo*, ameliorating histopathological lesions and deducing inflammatory infiltration in the lungs of mice infected intranasally with *M. bovis*. Overall, these results showed that 1E1 MAbs can decrease the colonization of *M. bovis* in the lungs and alleviate the lung lesions caused by *M. bovis*.

### Antibody-antigen contact interface residue prediction

**Fig 6:**
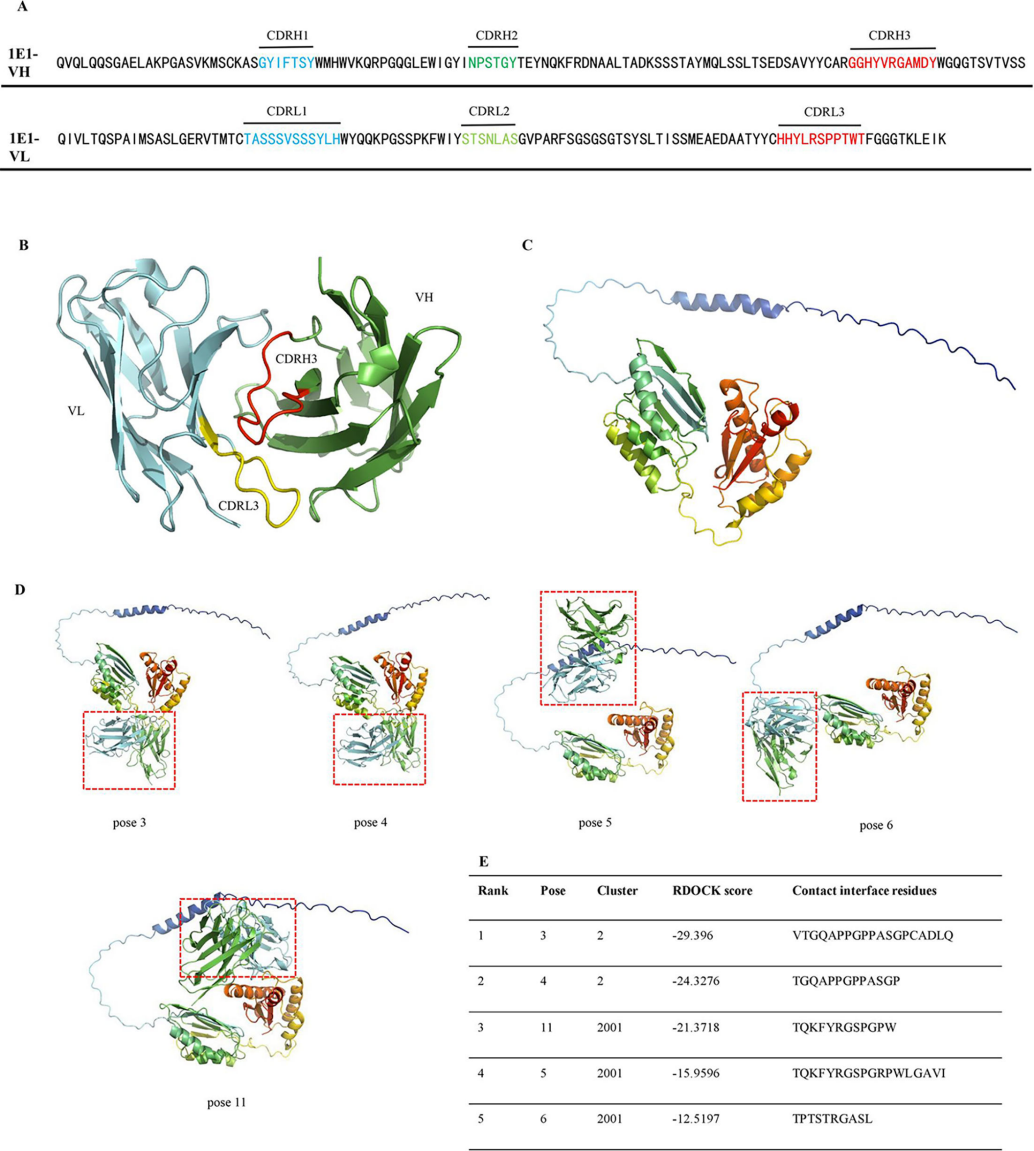
The contact liner epitope of 1E1. Using Discovery Studio (DS) to model Fv fraction. Utilized Alphafold three for modeling of OmpA. Pymol software was employed to represent the structure. (A) The antibody sequence analysis. The RNA from 1E1 antibody was prepared via TRIzol reagents (Invitrogen). The cDNA was synthesized with a cDNA synthesis kit (Enzyme, PR211-01). The antibody sequence analysis was completed by GENEWIZ and annotated refer to IMGT/V-QUEST (https://opig.stats.ox.ac.uk/webapps/sabdab-sabpred/sabpred/abodybuilder/). (B) Antibody Fv modeling with VH (green) and VL (gray) domains. The antibody Fv structure was modeled by DS. (C) Antigen structure model. The antigen structure model was predicted via AlphaFold 3. (D) Top five highest-ranked antibody-antigen complex poses, 3, 4, 11, 5, and 6. The antibody was shown in the red box. (E) Details of top highest-ranked, by RDOCK score, rigid-body docking poses.

The accessibility of an antigen epitope is a crucial factor for antibody binding. The 1E1 antibody sequence is shown in Fig. 6A. The antibody model was generated using DS 2019 with homology models as shown in Fig. 6B, and the monomer structure of OmpA was predicted by Alphafold three server and used for docking (Fig. 6C).

To analyze the antibody epitope, the antibody model and antigen structure were inputted into DS 2019 software. The results showed 2000 poses subjected to ZDOCK (rigid-body docking) with an angular step of 15° and a cutoff of 10 Å. All poses were filtrated based on the most core CDR3 region, producing 17 poses that contacted the CDR3 of 1E1 antibody. Subsequently, the 17 poses were optimized using the RDOCK (refined docked protein). The five highest-ranked poses and each pose׳s contacted interface residue of antigen are listed in Fig. 6D and E.

## DISCUSSION

The abuse of antibiotics can result in the emergence of multi-drug resistant Mtb, thereby posing significant challenges to the control of TB. The urgent need for an effective vaccine has accelerated the development of vaccines designed to combat the disease. The characterization of protective antibodies elicited by mycobacterial species represents a novel approach to the development of an optimal vaccine. In this study, we developed and characterized an antibody against OmpA both *in vitro* and *in vivo*. The antibody was observed to promote phagocytosis and intracellular killing of macrophages in a dose-dependent manner. Furthermore, the inhibitory effect of the antibody on growth was determined in *ex vivo*. Additionally, the efficacy of passive immunized 1E1 antibody before i.n. infection was evaluated in C57BL/6J mice, resulting in a 0.7 log reduction of bacterial lung load. Similarly, 1E1 MAb also exhibited an obvious therapeutic effect in mice, which reduced the lung burden and alleviated the histopathological lesions. These findings suggest that targeting the OmpA protein may offer a promising avenue for the development of novel therapeutic strategies against TB.

The use of MAbs provides a novel approach to addressing the challenges posed by tumors, inflammatory processes, and infectious diseases, due to their unique specificity and their lack of susceptibility to tolerance. In past years, the high cost of antibody development has greatly limited its extensive applications in the therapy of pathogenic infections. However, MAbs therapeutics for TB, benefiting from the advance of antibody development technology, are actively evolving in response to the increasing multi-drug resistance. In the past two decades, a few antibodies exhibiting effective mycobacterial growth that were inhibited both *in vitro* and *in vivo* have been reported. The antibody 9d8, specific to the capsular polysaccharide of Mtb, enhanced mice survival by approximately 30–60% (30). Balu et al. reported that the 2E9 antibody, targeting the 16 kDa α-crystallin, could reduce lung colonization and pathology in 2011 (31). Antibodies targeting mycobacterial surface proteins PstS-1 (26) or LpqH (27), isolated from TB patients and highly exposed individuals, also exhibited significant mycobacterial inhibition, indicating potential treatment value. Additionally, combining the MAbs and antibiotics may enhance therapeutic effects and reduce the required antibiotic concentration while preserving their efficacy. The therapeutic approach also reduces the dosage of antibiotics needed and lowers the emergence of drug-resistant Mtb (32, 33). As a result, these MAbs, which inhibit mycobacterial growth, hold cautious optimism for clinical settings in the years ahead.

The variable roles of the OmpA family of proteins, including drug resistance, acid tolerance, ion channel, and immune response in bacteria, have been extensively reported (34–37). In particular, a variety of pathogenic mechanisms was exhibited in OmpA-like proteins, reflected in multiple ways, such as adhesion, invasion, and host cell activation (38, 39). Furthermore, the high copy number and surface exposure of OmpA proteins generated an excellent therapeutic target for the immune system, in which the OmpA could be used as an activator. Because of the prevalence of antibiotics and the intracellular symbiotic nature of Mtb, the potential value of antibodies has been overlooked for a long time. Until recent years, the antimicrobial functions of antibodies in pathogenic bacteria were gradually revealed. The protective antibodies elicited by OMP in many pathogenic bacteria have been revealed in previous research (17, 18, 40–42). Among them, OmpA antibodies of *A. baumannii* and *E. coli* demonstrated a remarkable ability for anti-bacterial therapy, emphasizing the value of OmpA as a therapeutic target. Importantly, the OmpA of *M. bovis* produces a protective antibody characterized *in vitro* (Fig. 2G through I) and *in vivo* (Fig. 4D and 5D), indicating that the OmpA protein could be considered a candidate for anti-TB vaccine development in the future.

The prerequisite for antibody functioning is the accessibility of antigens. As previously reported, OmpA family proteins were extensively distributed in both gram-negative and gram-positive bacteria, anchored to cell walls through noncovalent interactions. This is an advantage for antibody accessible. The absence of a hydrophobic α-helix and the presence of a signal peptide and β-barrel structure classifies OmpA as a surface protein of Mtb, with the N-terminal anchored in the out membrane and the C-terminal exposed on the surface of Mtb (43). The results of bacterial ELISA showed variable binding abilities for BCG and *M. bovis* grown in medium with or without Tween-80 (Fig. 1D), implying that the antibody binding epitope is localized in the bacterial surface region. In the experiment, for pragmatic purposes, the BCG and *M. bovis* were cultured in medium with Tween-80. The addition of Tween-80 results in the loss of mycobacterial surface components including arabinose, glucose, mannose, arabinomannan, and phthiocerol dimycocerosate (44). The change in histological appearance of BCG and *M. bovis* might alter the extent of antigen exposure, which could either positively promote binding or negatively inhibit binding. The addition of Tween-80 might lead to the epitope folding targeted by 1E1 antibody, which decreases the antibody׳s ability to recognize the antigen. Additionally, antibody-antigen complex docking was used to analyze the contact residues of antibodies, with the top five highest-ranked poses shown in Fig. 6D. Among them, the interface residues contacted by pose 3 and pose 4 highly overlap in the region (196–212 aa) localized in C-terminal of OmpA, whereas the other poses contact regions close to N-terminal and are not readily bound by the antibody. These results illustrate the possible regions targeted by mAb 1E1. The actual epitopes recognized by the antibody 1E1 need to be further investigated. Antibodies obtained from persons with TB were observed to promote inflammasome activation, phagolysosomal fusion, and intracellular killing by macrophages (13). In this research, we identified the isotype of the 1E1 antibody as IgG2b, which has an obvious advantage in transfer through the placenta and protection of fetuses (45). Additionally, this isotype is the third most abundant in the bloodstream and plays an essential role in adaptive immune response. It actively participates in multiple anti-pathogen activities, including opsonization, complement activation, phagolysosomal fusion, inhibited intracellular growth, and antibody-dependent cell-mediated cytotoxicity (13, 46). As shown in Fig. 2A and B, the opsonizing role of the 1E1 antibody was demonstrated in a dose-dependent manner. Similarly, the increase in phagolysosomal fusion was revealed, as shown in Fig. 2C and D. The same decline in mycobacterial growth inhibition was observed in both BCG and *M. bovis* (Fig. 2F and G). These results are consistent with the characterization of antibody specific to Mtb.

In this research, a detailed investigation of the protective mechanism of the antibody was not conducted and awaits further study. Another antibody function-determining region is the constant domain, also called the Fc fraction. The Fc domain can affect not only affinity and avidity between Fab and antigen but also the recruitment of complement and innate immune cells to engage in its effector functions, such as antibody-dependent phagocytosis and antibody-dependent cytotoxicity (47). It has been demonstrated that it can directly modulate the physiology of intracellular pathogens in innate and adaptive immune responses (48). Apart from the Fc domain, the roles of T cells and MHC receptors remain to be revealed, as they play a critical role in adaptive immune responses. In addition, the broad-spectrum activity of the OmpA antibody is worth continuing to explore in the future, given that the OmpA protein has a wide range of homology in gram-negative species bacteria including *E. coli* and *A. baumannii* (Fig. S3A). Our whole bacterial ELISA has confirmed that the antibody possesses the potential to bind other bacteria bearing OmpA, such as nontuberculous mycobacterial species (Fig. S3B). However, the explicit cross-immunological protection must be assessed in a variety of animal models challenged with other bacteria bearing OmpA. Mycobacterial species are classic intracellular bacteria, with limited exposure to antibodies except during host cell-to-cell transfer. The use of antibodies engineered to couple Fab fragments of therapeutic antibodies against different antigens to form bispecific or trispecific antibodies may enhance the therapeutic effect of the antibodies, which is an interesting study in the future.

## Data Availability

Data will be made available on request.

## SUPPLEMENTAL MATERIAL

Supplemental tables and figures spectrum.02942-24-s0001.pdf Tables S1 to S3; Fig. S1 to S3.

ASM does not own the copyrights to Supplemental Material that may be linked to, or accessed through, an article. The authors have granted ASM a non-exclusive, world-wide license to publish the Supplemental Material files. Please contact the corresponding author directly for reuse.
