# Supplementary material for: Isolation and characterization of a protective monoclonal antibody targeting outer membrane protein (OmpA) against tuberculosis
Source: Microbiol Spectr. 2025 Feb 18;13(4):e02942-24. doi: 10.1128/spectrum.02942-24 (PMC11960079; doi:10.1128/spectrum.02942-24)
Supplement: Supplemental tables and figures — Tables S1 to S3; Fig. S1 to S3. [file spectrum.02942-24-s0001.pdf]

Supplementary Tables:

Table S1. Scoring criteria for evaluating the pathological changes in lung and spleen architecture.

**Lung**			
**Inflammation**		**Alveolar septal thickening**	
0	None	0	None
1	Sparse inflammatory infiltrate	1	Slight thickening
2	Mild inflammatory infiltrate	2	Mild thickening
3	Multiple inflammatory lesions	3	Moderate thickening
4	Severe inflammatory infiltrate	4	Severe thickening
**Bronchial mucus exudation**			
0	None		
1	Slight mucus exudation		
2	Mild mucus exudation		
3	Moderate mucus exudation		
4	Severe mucus exudation		

Table S2. The pathological score for changes in lung architecture from *M.bovis*-infected mice of the prevention group.

Organs	Items	PBS	1E1	MGO53
Lung	Inflammation	1	1	1
	Alveolar septal thickening	0	0	0
	Bronchial mucus exudation	1	1	1
	Stratification	+	+	+

Table S3. The pathological score for changes in lung architecture from *M.bovis*-infected mice of the therapeutic group.

Organs	Items	PBS	1E1
Lung	Inflammation	2	1
	Alveolar septal thickening	2	1
	Bronchial mucus exudation	1	1
	Stratification	++	+

Figure S1

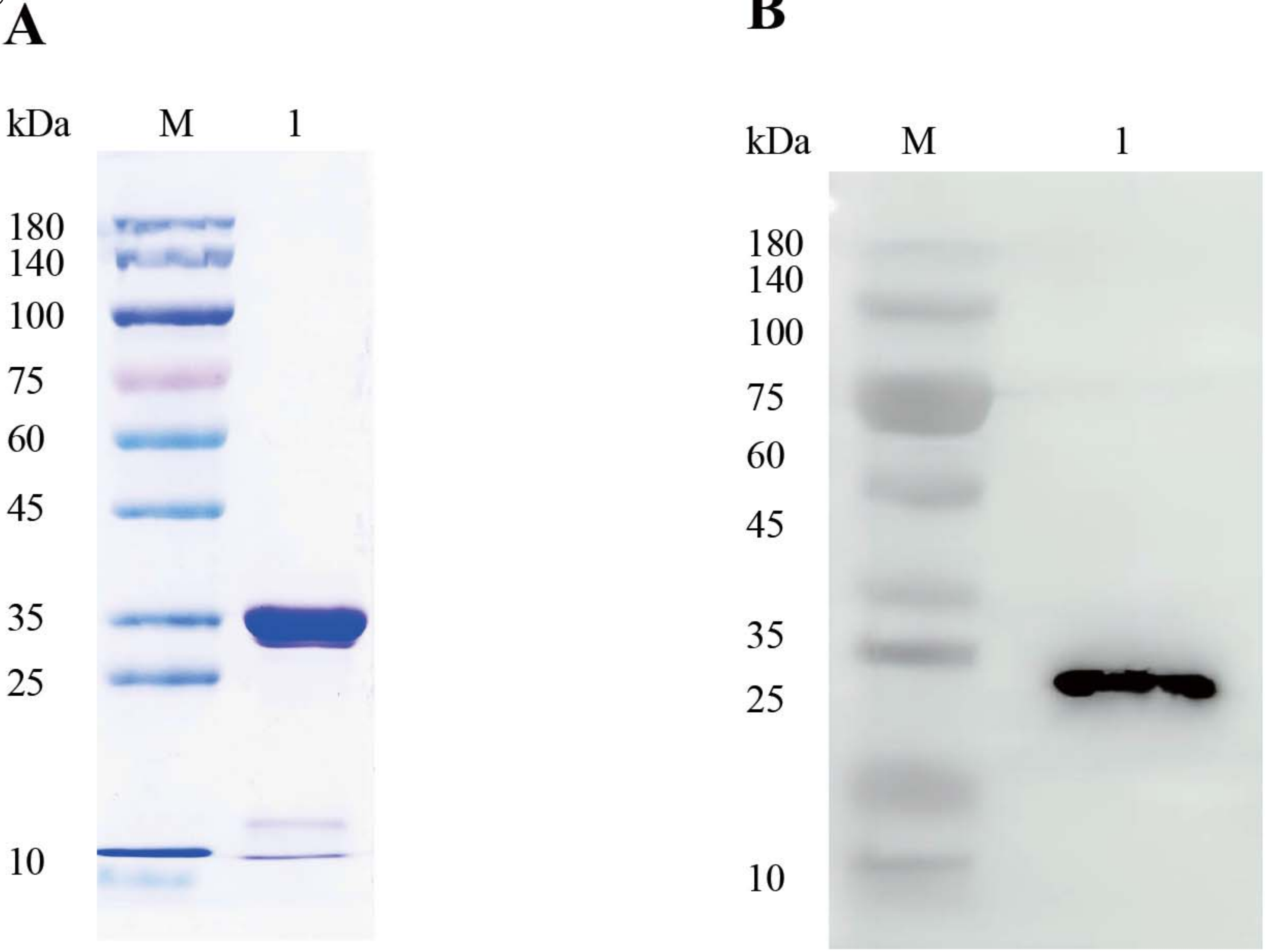
A
kDa
M
1
180
140
100
75
60
45
35
25
10
B
kDa
M
1
180
140
100
75
60
45
35
25
10

Figure S2

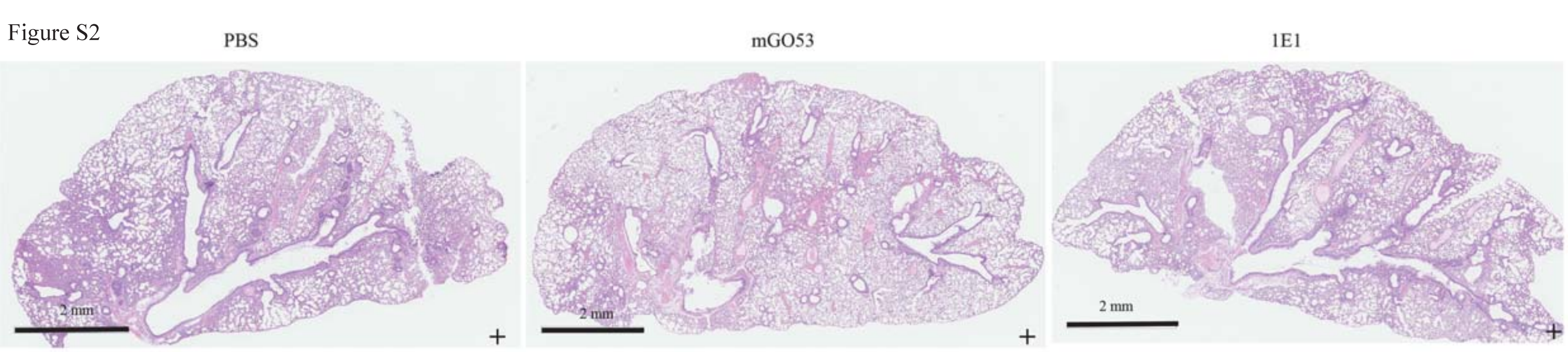

Figure S3

A

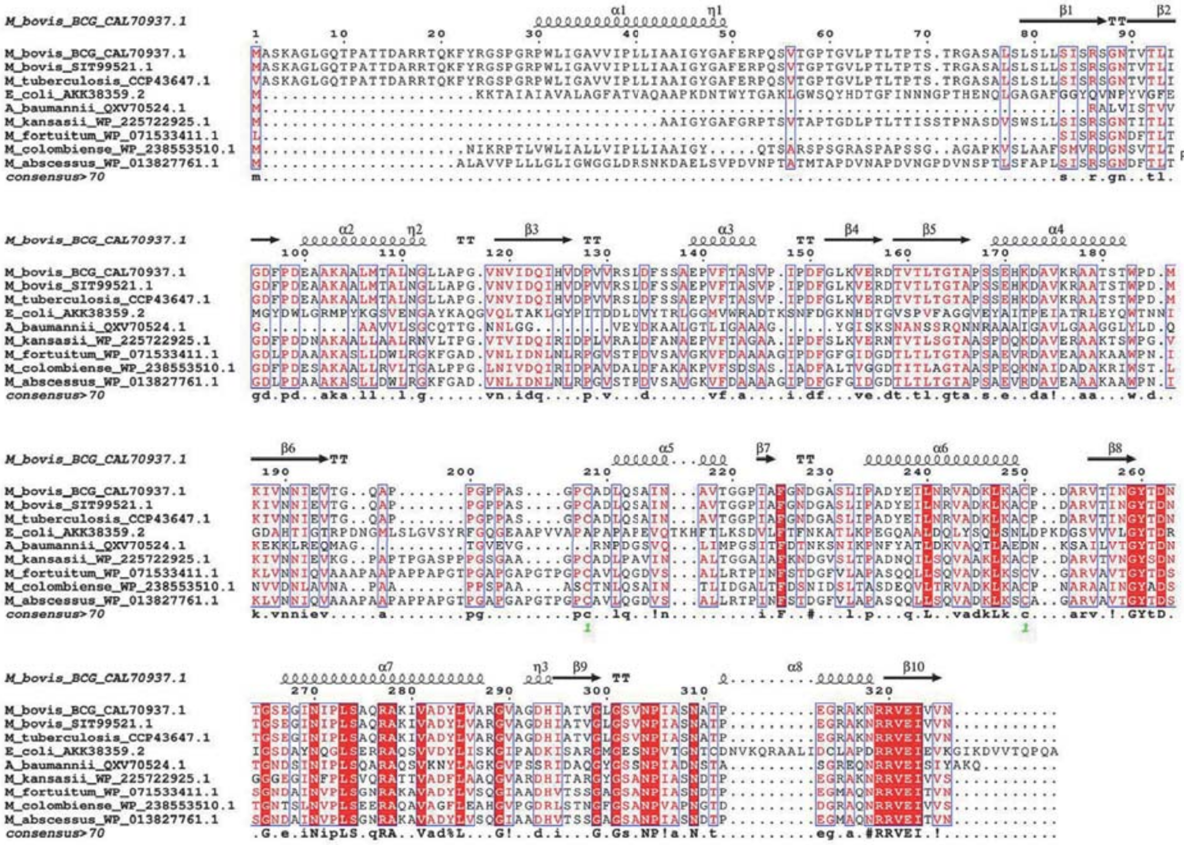
M_bovis_BCG_CAL70937.1
M_bovis_SIT99521.1
M_tuberculosis_CCP43647.1
E_coli_AKK38359.2
A_baumannii_QXV70524.1
M_kansasii_WP_225722925.1
M_fortuitum_WP_071533411.1
M_colombiense_WP_238553510.1
M_abscessus_WP_013827761.1
consensus>70

B

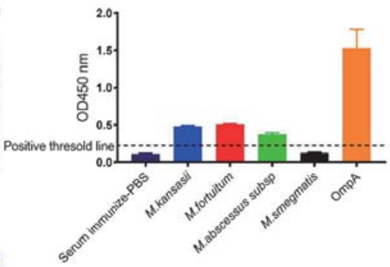
OD450 nm
2.0
1.5
1.0
0.5
0.0
Positive thresold line
Serum immunize-PBS
M.kansasii
M.fortuitum
M.abscessus subsp
M.smegmatis
OmpA

Supplementary Figure Legends:

Supplementary Figure S1: The preparation and purification of OmpA. A, SDS-PAGE analysis demonstrates the purification of OmpA. B. Western-blot analysis demonstrates strong reactivity of purified OmpA protein with His antibody.

Supplementary Figure S2: Representative pathological images of lung from *M.bovis*-infected mice using HE staining. The top, scale bars show 2 mm; the Bottom, scale bars show 100 µm. The image was annotated using the Aperio ImageScope (Leica Biosystems). The extent of tissue damage was scored in the bottom right corner of each Figure, with the symbol "+" indicating a progressive increase in severity.

Supplementary Figure S3: Multiple alignment of OmpA in different bacterial strains and cross-reactivity of 1E1 against NTM strains. (A) The homologous alignment of OmpA amino acid sequence from different strains. All bacterial including *Mycobacterium bovis* BCG (*M_bovis BCG*), *Mycobacterium bovis* AF2122-97 (*M_bovis*), *Mycobacterium tuberculosis* H37Rv (*M_tuberculosis*), *Escherichia coli* APEC O2-211 (*E_coli*), *Acinetobacter baumannii* ATCC 19606 (*A_baumannii*), *Mycobacterium kansasii* ATCC 12478 (*M_kansasii*), *Mycolicibacterium fortuitum subsp. fortuitum* DSM 46621 (*M_fortuitum*), *Mycobacterium colombiense* 10B (*M_colombiense*), and *Mycobacteroides abscessus* (*M_abscessus*). The consensus has 70% similarity of the sequence. (B) The cross-reactivity of antibody with NTM strains was assessed via whole bacterial ELISA. The x-axis represents 3 NTM strains and *M. smegmatis* $mc^2155(10^7$ CFU/well) coated on 96-well plates and reacted with 1E1 antibody (1:5000). The serum from immunized-PBS was used to negative control. Positive control was established by reacting to the purified OmpA protein with Mab. *M.kansasii*, *Mycobacterium kansasii*; *M.fortuitum*, *Mycobacterium fortuitum*; *M.abscessus subsp*, *Mycobacterium abscessus subsp*; *M. smegmatis, Mycobacterium smegmatis*.
